# The heterocellular heart: identities, interactions, and implications for cardiology

**DOI:** 10.1007/s00395-023-01000-6

**Published:** 2023-07-26

**Authors:** Achim Lother, Peter Kohl

**Affiliations:** 1 Institute of Experimental and Clinical Pharmacology and Toxicology, Faculty of Medicine, University of Freiburg, Albertstr. 25, 79104 Freiburg, Germany; 2 Interdisciplinary Medical Intensive Care, Faculty of Medicine, Medical Center—University of Freiburg, University of Freiburg, Freiburg, Germany; 3 Institute for Experimental Cardiovascular Medicine, Faculty of Medicine, University Heart Center, University of Freiburg, Freiburg, Germany; 4 CIBSS Centre for Integrative Biological Signalling Studies, University of Freiburg, Freiburg, Germany

**Keywords:** Non-myocyte, Macrophage, Fibroblast, Signalling, Single-cell sequencing, Drug-targeting

## Abstract

The heterocellular nature of the heart has been receiving increasing attention in recent years. In addition to cardiomyocytes as the prototypical cell type of the heart, non-myocytes such as endothelial cells, fibroblasts, or immune cells are coming more into focus. The rise of single-cell sequencing technologies enables  identification of ever more subtle differences and has reignited the question of what defines a cell’s identity. Here we provide an overview of the major cardiac cell types, describe their roles in homeostasis, and outline recent findings on non-canonical functions that may be of relevance for cardiology. We highlight modes of biochemical and biophysical interactions between different cardiac cell types and discuss the potential implications of the heterocellular nature of the heart for basic research and therapeutic interventions.

## Introduction

The heterocellular nature of the heart has been known to researchers since the early days of histological studies, and our knowledge of the cellular composition of the heart and of the functions of the different cell types has steadily increased in complexity. Quantitative analyses of the heterocellular make-up of the heart moved from histology [3] and electron microscopy [125] of native tissue towards flow cytometry of isolated cardiac cells [14]. Unsurprisingly, quantitative analyses of cell abundance, based on tissue sections or isolated cells differed. In recent years, the wider availability of RNA sequencing (RNA-seq) techniques has allowed a different and more (though not yet fully) unbiased approach to cell type characterization [29, 185].

**Fig. 1:**
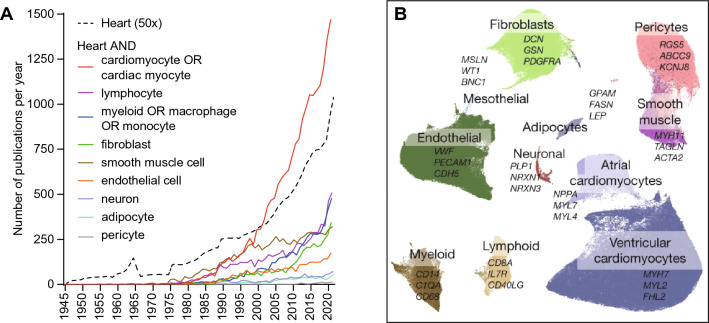
The heterocellular heart. **A** Number of publications per year included in PubMed between 1945 and 2021 that refer to the heart and one of the specified cell types in the title or abstract, compared to publications that refer to the heart only (divided by a factor of 50 for better visibility). **B** Uniform manifold approximation and projection (UMAP) of 11 cell types of the adult human heart as identified by scRNA-seq (from [102] under Creative Commons Attribution 4.0 International License, http://creativecommons.org/licenses/by/4.0/)

For the characterization of cell properties, an important milestone was the development of suitable cell culture techniques from the 1960s onwards, which enabled researchers to study cardiac cell function in vitro [66, 155]. Since the 1990s, transgenic animal models with gene overexpression or deletion under the control of cell-selective promoters have been applied to modulate cellular function in vivo [43, 140, 190]. The growing interest in cardiac cell types [62] is reflected by a substantially growing number of scientific publications. While cardiomyocytes, as the prototypical cell type of the heart, remain the most frequently studied individual cell type, the combined number of published papers per year focused on cardiac immune cells or fibroblasts is comparable (Fig. 1A).

The dynamic changes in the heart’s cellular composition and the interactions between different cardiac cell types in development [10, 43, 159] and disease [55, 72, 113, 130, 134, 141, 176, 186, 196, 199, 205] have been excellently reviewed elsewhere. Here, we provide an overview of cardiac cell identities, describe their canonical functions during homeostasis, and provide examples for more recently discovered ‘non-canonical’ functions that may be of relevance for cardiology. We summarize biochemical and biophysical interactions between different cardiac cell types under steady-state conditions, which have received limited attention so far, and discuss potential implications of the heterocellular nature of the heart for basic research and therapeutic interventions.

## Identities

### Cell types, subtypes, and cell states in the adult heart

When discussing cardiac cell types and their functions, we first need to define cellular identities. However, this is far from trivial. Traditionally, cell types (e.g. cardiomyocytes) and specialized subtypes (e.g. ventricular cardiomyocytes, atrial cardiomyocytes, pacemaker cardiomyocytes) were annotated according to their morphological phenotype, the presence of certain surface antigens, their function, and/or later genetic lineage tracing. The rise of single-cell RNA-seq (scRNA-seq) technologies and the accompanying possibility of making ever more subtle distinctions, but also the user-dependence of RNA-seq data analysis, presentation and interpretation, have refuelled the ongoing debate on how to define a ‘cell type’ [123, 195, 218]. For scRNA-seq, RNA of each cell is converted into complementary DNA (cDNA) by reverse transcription, individually barcoded by the insertion of a short nucleotide sequence, amplified, and processed for sequencing [185]. Bioinformatics analyses of the resulting raw sequencing data are then used to determine gene expression profiles of individual cells. Similarity of cells is defined according to the similarity in their gene expression, and this information is then used to define their positions in a multidimensional virtual space. To reduce dimensionality, genes that show high correlation in expression are grouped into modules [195]. Depending on how far the dimensionality of sequencing data is reduced, each cell can be assigned to a larger or smaller cluster of cells with similar gene expression profiles (Fig. 1B). This approach has two important implications: first, the number of cell types detected by this kind of analysis depends on the extent of dimensionality reduction prescribed in the analysis. Second, newly defined cell types, inferred from transcriptome analysis, require validation by a different technique.

Since changes in gene expression can be highly dynamic within one and the same cell population, a different transcriptomic signature does not necessarily define a distinct cell type. Differences that are determined by a cell’s local environment, or that occur in response to stimuli—but which do not alter cell identity—are often referred to as ‘cell states’ [123, 195]. Thus, one cell type can have multiple states—which, in the absence of agreed standards on defining cell types—has given rise to large variability in the way RNA-seq based information is presented visually and conceptually. Their identification generally requires a priori knowledge about the relevant biology, which is not always available when exploring hitherto unknown biological processes. Furthermore, gene expression data can be integrated with information from in situ hybridization or targeted barcoding to localize cells in the tissue, providing spatial—albeit thus far largely two-dimensional—information [83, 100]. Finally, epigenetic information such as chromatin accessibility can be included in the analysis to discriminate cell types and states [156, 187, 220]. Both scRNA-seq and single nucleus RNA-seq (snRNA-seq) techniques are used by researchers world-wide, including large international consortia, aiming to identify and spatially map all cell types in the human body [165].

In 2016, first reports on scRNA-seq of the developing mouse heart were published [40, 98] and since then, the technology has spread quickly in the cardiovascular field [28, 53, 60, 73, 99, 141, 166, 181, 188], right through to spatio-temporally resolved RNA-seq [10, 95, 111]. For technical reasons that are discussed in more detail below, nuclei instead of cells have been used in several studies for snRNA-seq of the heart [60, 73, 188]. Transcriptomics studies have described a large variety of cell types in adult human and rodent heart tissue, each of them with different subtypes and states. These include well-known players such as cardiomyocytes, endothelial cells, smooth muscle cells, fibroblasts, and myeloid or lymphoid immune cells. Other cardiac cell types, such as pericytes, adipocytes, neurons or Schwann cells, and their roles in physiology or pathophysiology of the heart, are also gaining increasing interest. An overview of commonly used cell type marker genes or surface antigens is provided in Table 1.

**Table 1:** Main cell populations and their functions in the healthy adult heart

Cell type	Main function in the healthy adult heart	Commonly used identifying markers	References
Cardiomyocytes		PLN, PCM-1	[59, 102]
Ventricular	Heart contraction	αMHC	
Atrial	Heart rhythm, heart contraction	MYL7	
Endothelial cells	Regulation of vascular contraction Trafficking of cells and metabolites	CDH5, PECAM-1	[102, 105, 140, 141]
Smooth muscle cells	Vascular contraction (arteries, arterioles)	MYH11, ACTA2, TAGLN	[25, 102, 184]
Pericytes	Vascular contraction (capillaries)	NG2, PDGFRβ	[11]
Fibroblasts	Extracellular matrix formation	PDGFRα, TCF21, DDR2	[84, 102, 152, 192]
Myofibroblasts		ACTA2	
Leukocytes		CD45	
Myeloid	Phagocytosis of cellular debris, immune surveillance	F4/80, CD11b	[189]
Lymphoid	Adaptive immune response	IL7R	[73]
Adipocytes	Energy supply, mechanical protection	LEP	[7]
Neurons	Sympathetic and parasympathetic regulation of heart rate and contraction	NRXN1, NRXN2	[102]
Schwann cells	Insulating nerve fibres	PLP-1	[74]
Mesothelial cells	Quiescent	WT-1	[26, 102, 159]

*αMHC* α myosin heavy chain, *ACTA2* actin α 2, *CD45* cluster of differentiation 45, *CD11b* cluster of differentiation 11b, *CDH5* cadherin 5, *DDR2* discoidin-domain receptor 2 *IL7R* interleukin 7 receptor, *LEP* leptin, *MYH11* myosin heavy chain 11, *MYL7* myosin light chain 7, *NG2* neuron–glial antigen 2, *NRXN1*/2 neurexin 1/2, *PCM-1* pericentriolar material 1, *PDGFRα/β* platelet-derived growth factor receptor α/β, *PECAM-1* platelet endothelial cell adhesion molecule-1, *PLN* phospholamban, *PLP-1* Proteolipid protein 1, *TAGLN* transgelin, *TCF21* transcription factor 21, *WT-1* Wilms’ tumour 1

The relative cell numbers of each population in the myocardium are still a matter of debate. Single-cell preparations are affected by differences in cell viability, caused by the techniques used for tissue disruption, cell isolation, and sorting. Early flow cytometry-based analyses of single-cell preparations of adult murine myocardium reported 56% cardiomyocytes (identified by α myosin heavy chain expression), 27% fibroblasts (discoidin-domain receptor 2; DDR2), 10% smooth muscle cells (α smooth muscle actin; αSMA), and 7% endothelial cells (platelet endothelial cell adhesion molecule-1, generally referred to as CD31) [14]. Note that these percentages relate to ‘cells identified and sorted’, not ‘cells in situ’, as is evident from the fact that they add up to 100% for just 4 of the many cell types known to be present in cardiac tissue. Slightly different results were obtained in snRNA-seq experiments on adult human ventricular myocardium, identifying 49% cardiomyocytes, 21% smooth muscle cells, 16% fibroblasts, 8% endothelial cells, and 5% immune cells [102]. In contrast, other studies using nucleus-labelling techniques found only about 30% cardiomyocytes in adult mouse and human hearts [15, 58], as had been indicated in earlier histomorphology-based studies on rat myocardium [125]. The current understanding suggests that an overwhelming majority of cardiac non-myocytes, identified using a combination of immunohistochemistry and flow cytometry, consists of endothelial cells (60% of non-myocytes), followed by mesenchymal cells including fibroblasts, smooth muscle cells, and pericytes (27% of non-myocytes) and leukocytes (9% of non-myocytes [148]. Numbers of rare cell types (neurons, Schwann cells, adipocytes) have not yet been reliably quantified. It is important to note that in addition to experimental aspects, other factors such as age or species may influence cardiac cell type composition. For instance, a modest decline of endothelial and mesenchymal cell numbers in the aging human heart has been reported [15]. In mice, the embryo-derived population of cardiac macrophages is gradually replaced by monocyte-dependent macrophages [120]. Both observations illustrate the dynamic turnover of cells in the adult heart.

### Canonical and non-canonical functions of cardiac cells

**Cardiomyocytes** are the main actors in cardiac pump function. So-called ‘working cardiomyocytes’ are comparatively large cells (> 10^4^ µm^3^) that are typically brick-shaped. They are connected end-to-end via intercalated discs, where desmosomes and fascia adherens junctions provide mechanical, and connexins electrical coupling. In addition, lateral mechanical connections link the contractile machinery at the z-disks to the extracellular matrix (ECM) as the main force-bearing structural element of the myocardium (a ‘deformable skeleton’), while lateral electrical connections occur mainly within the 4–6 cells thick layers of myocardium, referred to as ‘sheets’ or, more fittingly, ‘sheetles’.

Cardiomyocytes are densely packed with arrays of structural proteins that form the sarcomeres, membrane systems that enable Ca^2+^-mediated excitation–contraction coupling, and mitochondria. Cardiomyocyte contraction is a highly orchestrated process, involving multiple extra- and intracardiac feed-forward and feedback mechanisms [161]. Following cardiomyocyte depolarization, extracellular Ca^2+^ enters the cytosol via voltage-dependent Ca^2+^ channels and triggers further Ca^2+^ release from the sarcoplasmic reticulum. For relaxation, Ca^2+^ has to be removed from the cytosol. In steady-state, the amount released from intracellular stores is returned by the sarcoplasmic/endoplasmic reticulum calcium ATPase, while the initial ‘trigger-Ca^2+^’ is extruded back to the extracellular space via the sodium Na^+^/Ca^2+^ exchanger [17, 49]. The rapid and efficient activation of intracellular contractile units of a cardiomyocyte depends on transverse (T-) tubules, a network of membrane invaginations containing ion channels and transporters. The narrow and tortuous structure of the T-tubular system may impede diffusion and cause regional microdomains of different ion concentrations [171]. Similar to the heart as a whole that serves as a pressure-suction-pump in the circulation, T-tubules are squeezed by contracting cardiomyocytes, pushing and pulling extracellular fluid between T-tubular lumen and bulk extracellular space. This recently described mechanism accelerates T-tubular diffusion dynamics, which may be important in particular at high heart rates [94, 171].

Sarcomeres are highly organized structures composed of actin, myosin, and titin filaments, and a number of accessory proteins [202]. During cardiomyocyte contraction, myosin filament heads interact with actin filaments, forming so-called ‘crossbridges’ that govern force development and sarcomere shortening. This crossbridge cycling requires the hydrolysis of one ATP per power-stroke, leading to an immense flux of ATP that is reflected by a high mitochondrial density in cardiomyocytes [42, 121]. Cardiomyocyte mitochondria communicate with one-another [75], and they participate in Ca^2+^ handling [177, 179].

While cardiomyocytes from left or right ventricle share high similarity [102], atrial cardiomyocytes have a different phenotype, with distinct Ca^2+^-handling, contractile and electrophysiological properties [21, 128, 204]. Atrial cardiomyocyte sarcomeres contain different contractile protein isoforms, and while they develop less mechanical force, compared to ventricular myocytes [128], they contract faster [20, 127].

Working cardiomyocytes in the atria and ventricles are activated by a well-coordinated action potential wave, which originates from sino-atrial node pacemaker cells, and spreads through the atria, the atrio-ventricular node and His–Purkinje system, to the ventricular myocardium. The cells of sinus node and conducting system are specialized cardiomyocyte subtypes that show a distinct repertoire of ion channel expression and activity, which conveys upon them the spontaneous rhythmic depolarization that underlies pacemaking [204]. These cells are also cardiomyocytes, showing cross striations caused by sarcomeric arrangement of their contractile filaments, even if that had been questioned or overlooked in some of the early studies.

During pre-natal heart growth, cardiomyocytes show a high proliferation rate [215]. After birth, cardiomyocyte proliferation rate declines rapidly and remains at no more than about ~ 1% in adult human [51, 201]. When the cell cycle abrogates before cytokinesis, cardiomyocytes become multinucleated, polyploid, or both. The extent of bi- or multinucleated (human 26%, mouse 78%) and polyploid cardiomyocytes (human 58%, mouse 10%) in the adult heart varies between species [6, 15]. Postnatal growth of the adult heart is largely a function of cardiomyocyte hypertrophy, often associated with increased ploidy [69, 215]. Cardiomyocyte growth can be triggered by a variety of physiological or pathological stimuli, including mechanical and biochemical factors. For example, physiological stimuli caused by exercise or pregnancy can promote cardiomyocyte growth while maintaining or improving cardiac function, whereas pathological stimuli such as sustained volume or pressure overload (in valve disease of hypertension) can lead to hypertrophy with impaired function, altered metabolism, and dysregulated intracellular signalling [126]. Cardiomyocytes not only receive growth signals, but they may stimulate local blood vessel sprouting and innervation via secretion of growth factors such as vascular endothelial cell growth factor (VEGF) [134] or nerve growth factor [44].

**Endothelial cells** form the inner layer of the vasculature. They have distinct functions according to their localization and their association with different vascular beds. In the heart, capillary endothelial cells represent the largest cell population [102]. Other subtypes include arterial, venous, lymphatic, and endocardial endothelial cells [102]. All endothelial cells share high expression of cadherin 5 [102, 140] and platelet endothelial cell adhesion molecule-1 (which shows weak expression also in the hematopoietic lineage; [140]. Subspecification of endothelial cells is tightly regulated by the activity of transcription factors, in particular SOX (SRY-related HMG-box) and FOX (forkhead box) family members [112, 154].

Endothelial cells sense and respond to mechanical and biochemical stimuli [34, 41, 167]. Factors such as blood flow, mechanical stretch, and the interaction of cell membrane proteins with the extracellular environment influence transcription factor activity and gene expression in endothelial cells [143]. It has been proposed that the presence of pulsatile or laminar flow, and of high or low shear stress determine arterial versus venous endothelial cell differentiation [154]. Moreover, it has been shown recently that mechanical stress can induce endothelial-to-mesenchymal transition by activating the force-sensitive transforming growth factor β type I receptor kinase [115]. This represents an example of how cells can change their phenotype in response to external stimuli, taking different functions in homeostasis or patho/physiological remodelling [217].

Endothelial cells are the main source of nitric oxide in the heart, which via cyclic guanosine monophosphate (cGMP)-dependent signalling induces smooth muscle cell relaxation and vascular dilation [203]. Endothelin-1 (ET-1) from endothelial cells is a potent vasoconstrictor, primarily by acting on ET_A_ receptors on smooth muscle cells. On the other hand, autocrine ET-1 signalling, acting via ET_B_ receptors on endothelial cells, increases nitric oxide synthesis and inhibits ET-1 production in a negative feedback loop [203].

Cardiac capillary endothelial cells form a dense layer, with cells connected via tight junctions, which controls vascular permeability and trafficking of cells between blood and the surrounding cardiac tissue [47]. The high energy demand of cardiomyocytes demands high flux rates for oxygen and carbon dioxide, which benefits from a tight capillary network [134] with a capillaries-to-myocyte ratio of between 1.3:1 and 1.5:1 [90, 107]. During angiogenesis, a complex network of angiocrine factors such as VEGF guides endothelial cells to alter their state, proliferate, migrate, and form new capillaries [48].

**Smooth muscle cells** are a major constituent of coronary arterial and arteriolar walls. Their physiological function involves active changes in vessel cross-sectional area by contraction of circularly oriented cells, thereby controlling vascular resistance and—hence—regional blood flow (of note, the resistance R to flow is a fourth power inverse function of vessel radius r: R ~ r^−4^) [46, 104]. Efficient autoregulation of myocardial perfusion is of pivotal importance as it is positively correlated with contractile function [71]. In the healthy heart, arterioles are the primary site of flow regulation. Several autoregulatory mechanisms (endothelial, neural, metabolic, myogenic) adjust myocardial blood supply through effects on vascular smooth muscle cells [82]. In addition, smooth muscle cells respond to a number of paracrine and endocrine factors such as nitric oxide, endothelin-1, angiotensin II, aldosterone, or norepinephrine, secreted by cardiac cells or delivered through the circulation [46].

Traditionally, a contractile and a synthetic smooth muscle cell phenotype have been distinguished, though these are more likely to represent two ends of a continuum. Contractile smooth muscle cells are packed with (non-sarcomeric) myofilaments, which can be identified by expression of myosin heavy chain 11 or smooth muscle cell actin α 2; [25, 184]). Similar to cardiomyocytes, smooth muscle cell contraction depends on Ca^2+^ fluxes, but it is more than an order of magnitude slower [46].

Substantial plasticity of smooth muscle cells has been observed, including conversion to myofibroblast- or macrophage-like phenotypes [184]. Although smooth muscle cells are not thought to give rise to fibroblasts in the healthy heart [84], they modulate ECM remodelling by paracrine signalling [13, 150].

**Pericytes** are mesenchymal cells that share phenotypical similarities with smooth muscle cells and it has been suggested both cell types have a shared lineage, deriving from endocardial progenitor cells [30]. In pericytes, but not exclusively there, high expression of neuron–glial antigen 2 and platelet-derived growth factor receptor β can be detected [11]. Due to the lack of specific markers, pericytes and smooth muscle cells are usually distinguished by anatomical localization and sometimes summarized as mural cells [133, 148]. Pericytes are largely restricted to the microvasculature [9, 12, 27]. They discontinuously surround capillaries, forming circumferential and longitudinal processes that can contribute to vessel contraction, including contributions to post-ischaemic non-reflow phenomena [38, 70, 133].

Together with smooth muscle cells and pericytes, **fibroblasts** belong to the group of mesenchymal cells. They are characterized by high expression of platelet-derived growth factor receptor α, DDR2, and transcription factor 21 [84, 141, 192]. Cardiac fibroblasts may be distinguished by their localization (e.g. ventricular or atrial interstitial fibroblasts versus valvular fibroblasts), activation status (e.g. fibroblast, myofibroblast), or origin. While the vast majority of fibroblasts in the healthy heart is of epicardial origin, there is an ongoing debate whether other sources contribute to the cardiac fibroblast population after injury, including endothelial cells, bone marrow-derived cells, and other mesenchymal cells such as smooth muscle cells or pericytes [84, 141, 182, 191].

Fibroblasts secrete collagens and proteoglycans [55] and therefore they are traditionally regarded as the ‘cells that create and maintain... extracellular matrix’ [152]. However, over the last 30 years our view has substantially evolved, as it is evident that other cell types contribute to the ECM, while fibroblasts are now seen as much more versatile cells. Upon mechanical or biochemical activation, fibroblasts express contractile proteins such as αSMA and are then considered to be myofibroblasts [152]. In the context of cardiac lesion repair, up to four states have been suggested: resident fibroblast, activated fibroblast, myofibroblast, and matrifibrocyte, all with distinct functional properties [56]. Fibroblasts interact with one-another, and with other cell types, via multiple biochemical (cytokines, growth factors) [196] and biophysical cues (mechanical and electrical contacts) [24] to steer cardiomyocyte and non-myocyte functions as reviewed before and addressed in more detail below.

** Cardiac immune cells** comprise all major leukocyte classes of the innate and adaptive immune system, including myeloid cells such as macrophages, monocytes, dendritic cells, neutrophils, or mast cells, and lymphoid cells such as B cells and T cells. Each of these major cell populations contains several subtypes and states [102, 186, 189]. The cardiac macrophage population stems from different origins and can be distinguished from other leukocyte populations by combined surface expression of respective markers (CD45^+^, F4/80^+^, CD11b^+^) [189]. In steady-state, the population mainly consists of tissue-resident macrophages derived from yolk-sac progenitors, maintained by local proliferation, and a smaller, C–C chemokine receptor 2 (CCR2)^+^ macrophage population derived from circulating monocytes [50, 189]. After myocardial injury, CCR2^+^ monocytes make a major contribution to replenishment of the cardiac macrophage pool [67]. In the healthy heart, tissue-resident macrophages have essential roles in maintaining tissue homeostasis, as they are involved in ECM turnover and in the removal of cellular debris [189], which is of particular importance for organs that contain cells that are maintained for the life-time of an individual (see information on post-natal cardiomyocyte proliferation, above). Accordingly, experimental depletion of resident macrophages impairs the elimination of dysfunctional mitochondrial fragments from cardiomyocytes, leading to disturbed metabolism and ventricular dysfunction [131].

The heart is covered by the epicardium, a thin layer of **mesothelial cells**, and in particular in the perivascular areas by **adipocytes** [102]. The epicardium has been recognized as a multipotent cardiac progenitor tissue [26]. During cardiac development, Wilms’ tumour 1 expressing epicardial cells undergo epithelial-to-mesenchymal transition and give rise to multiple cell types, including fibroblasts, smooth muscle cells, and pericytes [26, 159]. In addition, epicardial cells steer coronary vessel formation via paracrine signalling [135]. In the adult heart, the epicardium is largely quiescent. However, it becomes re-activated upon injury and contributes to cardiac repair via cytokine signalling [159]. Whether epicardial progenitors are able, after tissue injury, to form cardiomyocytes or fibroblasts at significant numbers remains controversial [26, 84, 159].

Epicardial adipose tissue has several roles in cardiac physiology, such as energy supply and mechanical protection of the heart [7, 77]. In addition, there is increasing evidence for more complex interactions between adipocytes and myocardial cells. Via secreted factors such as adiponectin or leptin, adipocytes can influence cardiomyocyte Ca^2+^ cycling, metabolism, and redox state, and thereby alter contractility or contribute to arrhythmogenesis [7, 136]. This refers to circulating, visceral tissue-derived adipocytokines, but also to the paracrine action of epicardial adipocytes. In obesity, accumulation and inflammation of epicardial fat contribute to adverse cardiac remodelling, fibrosis, and arrhythmia (as recently reviewed elsewhere [77, 137]). Epicardial adipose tissue is most abundant in the atrio-ventricular and the interventricular sulcus, surrounding the coronary arteries [77]. More recently, a population of cardiac adipocytes has been identified that derives from cardiac mesenchymal cells and is located in subendocardial myocardium [81]. Whether these adipocytes have distinct functions remains to be explored. In diseases such as atrial fibrillation, fibro-fatty infiltration may contribute to electrical isolation of myocardial tissue regions, affecting action potential conduction and, potentially, facilitating the onset of arrhythmias [39, 52, 110].

Heart function is tightly controlled by the cardiac autonomous nervous system. Sympathetic and parasympathetic **neurons** located in ganglia of the sympathetic chain or in epicardial plexus form postganglionic nerve fibres reaching the myocardial tissue and modulating heart rate and contractility [5, 216]. In addition to this extrinsic part, the heart contains a large number of neurons forming an intrinsic cardiac nervous system. High-resolution imaging with subsequent 3D modelling revealed the majority of neurons to be localized in a compact region posterior of both atria with a dense network of neuronal processes reaching distant cardiac tissue [1]. Others suggested additional neuronal bodies within the ventricular myocardium [102]. Cardiac nerves also include **Schwann cells** and perimysial fibroblasts [74]. Their roles are assumed to be similar to those in non-cardiac nerves, although development, regulation, and remodelling of intracardiac nerves during aging and/or disease [174] are underinvestigated, beyond anatomical characterization.

Each cardiomyocyte is considered to be targeted by several neuronal processes via multiple neurotransmitter release sites [216]. More recently it has been reported that the cholinergic transdifferentiation of sympathetic neurons that occurs after myocardial injury may harmonize action potential durations and thereby reduce the risk for ventricular arrhythmia [209]. In addition, sympathetic neurons may, via direct cell–cell interactions (neuro-cardiac junctions), promote cardiomyocyte growth [147], while cardiomyocytes, via neuronal growth factor release, may help to sustain local innervation [44]. Thus, it is becoming increasingly clear that the intracardiac nervous system is subject of highly dynamic bidirectional neuro-muscular cross-talk.

## Interactions

**Fig. 2:**
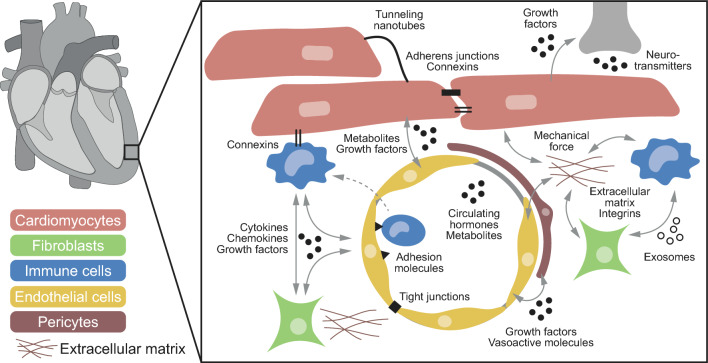
Heterocellular interactions in the healthy heart. Cell communication in the heart involves ‘biochemical’ and ‘biophysical’ cues. Secreted factors that act in a paracrine or autocrine manner include cytokines, chemokines, or growth factors. These factors may be secreted to the extracellular space, ‘wrapped up’ in exosomes, be transported via tunnelling nanotubes, etc. In addition, metabolites may act as signalling molecules. Cardiac cells are responsive to endocrine factors and neurotransmitters that may derive from the circulation or intracardiac nerve endings. Biophysical interactions involve direct mechanical cell–cell coupling via adherens junctions or tight junctions, as well as indirect mechanical coupling such as via extracellular matrix/integrins connections. Electrical coupling in the heart typically involves connexins, a class of transmembrane proteins that forms gap junctions between cells

Cell communication in the heart involves different modes of signal exchange, including ‘biochemical’ and ‘biophysical’ cues (Fig. 2). Biochemical signalling may occur in an autocrine (sender and receiver are the same cell) or paracrine way (sender and receiver are separate cells). Secreted factors include various classes of molecules such as cytokines, chemokines, or growth factors. These molecules typically act as ligands at membrane-bound or intracellular receptors of the target cell [162]. They may be secreted to the extracellular space, ‘wrapped up’ in exosomes, or transported between cells, for example via tunnelling nanotubes. In addition, metabolites such as nitric oxide, carbon dioxide, or reactive oxygen species can act as signalling molecules. Finally, cardiac cells are responsive to endocrine factors and neurotransmitters such as endothelin-1 or noradrenalin that may derive from the circulation, intracardiac nerve endings, and/or be produced by cardiac cells. Biophysical interactions involve direct mechanical cell–cell coupling via adherens junctions or tight junctions, as well as indirect mechanical coupling via extracellular matrix/integrin connections, through extracellular and vascular fluid mediated forces, as well as tissue shear, twisting, etc. Electrical coupling in the heart typically occurs through connexins, a class of transmembrane proteins that forms gap junctions between neighbouring cells, although other electrical interactions (ephaptic, capacitative) may be of functional relevance as well [180] (Fig. 2).

To enumerate all individual forms of cell communication in the heart would be an immense undertaking that is beyond the scope of a single review article. Instead, we summarize key examples of cell–cell interactions across a range of cell types and focus in the following on more targeted and in-depth analyses of exemplary new facets of heterocellular cross-talk that form potential areas for further research and therapeutic intervention.

### Biochemical cell interactions in the heart

Transcriptomic data from five cardiac cell types (cardiomyocytes, endothelial cells, fibroblasts, monocytes, macrophages) predicted more than 6000 possible heterocellular ligand–receptor interactions in healthy adult mouse heart [106]. Similar findings were obtained by analysing scRNA-seq data of non-myocytes [182], with fibroblasts accounting for the greatest proportion of potential heterocellular signalling pathways in both studies [106, 182].

Heterocellular biochemical signals in steady-state conditions are thought to be critical for forming and maintaining the cardiac microenvironment. For example, endothelial cells from different organs have highly specialized functions. Capillary endothelial cells in the kidney form a fenestrated endothelium, allowing renal filtration, while endothelial cells in the brain form a dense monolayer and express specific junctions and transporters that maintain the blood–brain barrier [154]. In the heart, endothelial cells appear to adapt to the high oxygen and energy demand of cardiomyocytes, which is reflected by a high capillary density and nutrient transport capacity [154]. Cardiomyocytes largely depend on fatty acids as their energy source, and cardiac endothelial cells, in contrast to endothelial cells from other organs, show high expression of fatty acid binding protein 4 (*Fabp4*), CD36, and other proteins that facilitate fatty acid uptake from the circulation [35, 80, 105, 132, 183]. It has been suggested that vascular endothelial growth factor B (VEGFB), acting on the VEGF receptor 1, is a key regulator of fatty acid transport. VEGFB is highly expressed in tissues which use fatty acids as an energy source, including the heart [65], indicating cardiomyocyte–endothelial cell cross-talk. Several studies showed that ablation of VEGFB in mice decreases the expression of fatty acid transport proteins, impairing fatty acid uptake [65, 109, 114]. However, there are conflicting data from another study using VEGFB deletion that could not reproduce that phenotype [89]. The transcription factors MEOX2 and TCF15 cooperatively regulate CD36 expression in endothelial cells, independently of VEGFB [35]. MEOX2/TCF15 haplodeficiency decreases CD36 expression, diminishes fatty acid uptake, and impairs left ventricular function [35]. Whether this represents an intrinsic pathway controlling fatty acid transport in cardiac endothelial cells, or is a response to yet undefined external signalling, remains to be explored.

Another example for heterocellular interactions that until recently were underestimated, is the role of pericytes in cardiac physiology and pathophysiology [12]. Pericytes are critical for vessel stability and the integrity of the capillary wall. During early vessel formation, as well as in neo-angiogenesis, sprouting endothelial cells release platelet-derived growth factor β (PDGFβ) which attracts pericytes [101]. During vessel maturation, pericytes in turn act on endothelial cells via paracrine angiopoietin-1 signalling, which regulates vessel permeability and, in a feed-forward mechanism, induces the expression of PDGFβ—thereby further enhancing pericyte attraction [27]. In addition to their effects on endothelial cell junctions, pericytes form a second, subendothelial vessel barrier. The endothelial cell-derived macrophage migration inhibitory factor decreases pericyte contractility, allowing transmigration of leukocytes into the surrounding tissue [142]. Pericyte contraction not only controls capillary permeability but also (re-)perfusion. Hyperemia induces pericyte contraction, leading to capillary derecruitment [117]. This can contribute to the fine-tuning of local tissue perfusion and energy supply. Recently, an elegant study demonstrated ATP-sensitive electrical feedback signalling through gap junctions from cardiomyocytes to microvascular cells, regulating pericytes, smooth muscle cell contraction, and blood flow [221]. After acute myocardial infarction, pericyte contraction may contribute to what is called the ‘no reflow’ phenomenon [116, 133]: despite successful re-opening of the occluded coronary arteries, tissue perfusion remains impaired. This involves capillary destruction, followed by myocardial haemorrhage and oedema, and impaired vasomotion [70]. Application of vasodilators such as adenosine may reverse capillary constriction, but also act on arterioles and induce unwanted hypotension [133]. Interestingly, deletion or pharmacological inhibition of G-protein coupled receptor 39 prevents pericyte contraction and improves tissue reperfusion in mice [116], thus representing a potentially more selective therapeutic approach compared to currently used vasodilators [38].

### Biophysical cell interactions in the heart

Biophysical cross-talk of cardiomyocytes with one-another and with the ECM is part of textbook-level knowledge in terms of electro-mechanical organization of cardiac function [146], and increasingly also in terms of other processes such as mechano-electric or mechano-chemical feedback [79].

Heterocellular biophysical cross-talk, in contrast, had long been ignored [91]. This is surprising, given that already the very first cardiac cell culture studies over 5 decades ago indicated that non-myocytes can form electrically conducting bridges between cardiomyocytes [61]. Heterocellular electrical coupling was subsequently confirmed using structured in vitro models, which showed that fibroblasts can passively conduct excitatory electrical signals over 300-μm gaps between groups of rat ventricular cardiomyocytes [57]. Confirmation of connexin-mediated electrotonic coupling in native myocardium became possible with the advent of optogenetic targeting of membrane potential reporters to non-myocytes [160], which established the presence of heterocellular electrotonic coupling between cardiomyocytes and non-myocytes, presumably fibroblasts, in cardiac lesions after ablation and myocardial ischaemia [160, 172]. Such heterocellular coupling allows electrical conduction along stretches of myocardium, which may not necessarily contain uninterrupted strands of cardiomyocytes [92]—a principle that has been used to design heterocellular tissue constructs that can bridge atrio-ventricular conduction upon destruction of the atrio-ventricular node [32].

In addition to cardiomyocyte–fibroblast electrical coupling in myocardial lesions or engineered tissue strands, it was recently discovered that this mode of signalling also is present between cardiomyocytes and macrophages in healthy murine myocardium [76]. The presence of connexin hemichannels and their relevance, both for heterocellular electrical coupling [93] and for the function of individuals cells (such as fibroblasts [93], pericytes [139] or endothelial cells [153], to name but a few), is ill-investigated and forms an exciting area for further study.

While electrotonic coupling affects cells in the immediate proximity, and with a ‘speed of conduction’ in the 10^0^ m/s range, mechanical coupling connects ‘all’ cells of the heart, over long distances, and essentially with the speed of sound (10^2^ m/s). Given that all cardiac cell types are mechano-sensitive, this offers vast potential for mechanically induced heterocellular signalling effects, including links to chemical signalling, such as via nitric oxide [19], ROS [210], micro-RNA [163] or calcium [78], to name but a few, potentially going full circle to effects on cardiac mechanical [85] and electrical function [145], or cardiomyocyte structure [214].

In addition to the general exposure of all cells to the dynamic cardiac mechanical environment, and to the effects of this on cell function, there are novel structural links emerging, whose relevance for biophysical signalling remain to be explored. This includes perhaps counter-intuitive and cardiac disease-modulated projections of ECM-associated collagen fibres into the T-tubular system of cardiac myocytes [36], stretch- and contraction-induced advection of T-tubular content that may contribute to sustenance of ion homeostasis in this crucial cell compartment [171], as well as the formation of heterocellular tunnelling nanotubes [94, 160] whose relevance for heterocellular signalling is unknown in the heart.

## Implications…

### …for basic research

**Fig. 3:**
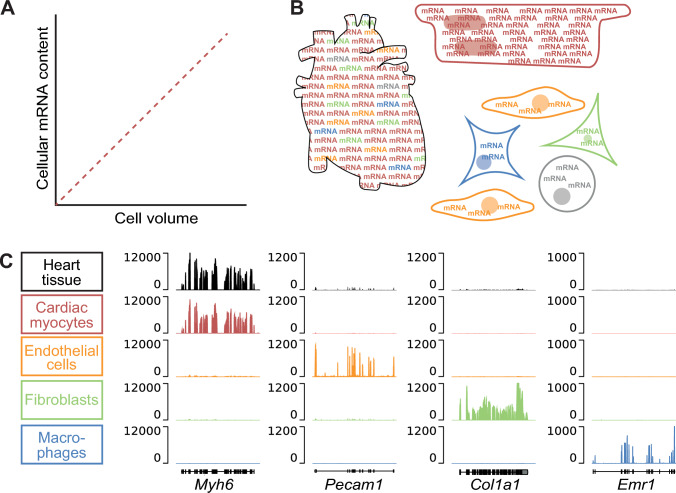
Gene expression in heart tissue compared to cardiac cells. **A** Cellular mRNA content is approximately linearly correlated with cell volume. **B** As cardiomyocytes are large cells that, in contrast to their numerical share, account for most of the total cardiac cell volume, they dominate mRNA expression in heart tissue. Conversely, mRNA from smaller and/or less abundant cell types is underrepresented in cardiac tissue mRNA pool analyses. **C** mRNA expression (fragments per kilobase of transcript per million mapped reads) in isolated cardiac cells, compared to whole heart tissue (modified from [106] with permission)

The heterocellular nature of the heart has important implications for cardiology and cardiovascular science, reaching from basic research to clinical application. In basic research, one needs to consider that the different cell types of the heart have distinct cellular, biochemical, and mechanical properties, adapted to their highly specialized functions. This implies that studying heterogeneous cells requires cell type-specific approaches. For instance, it is more and more accepted that gene expression and epigenetic analysis should be performed on the cell population of interest and not rely on bulk tissue data [144]. Gene expression analyses from bulk heart tissue will represent a mixed pool of mRNA from different cell populations. The mRNA content of a cell is approximately linearly correlated with cell volume (Fig. 3A; [86]). Cardiomyocytes are large cells, occupying a tissue volume that is disproportionate to their numerical share: they account for most of the tissue volume and thus mRNA in the heart (Fig. 3B). Conversely, mRNA from smaller and/or less abundant cell types is underrepresented in the tissue mRNA pool [105, 106]. As the majority of genes is expressed in more than one cell type, differential mRNA expression in one cell type may be masked by up- or down-regulation of the same gene in another cell type [106] (Fig. 3C). In addition, the make-up of cardiac cell populations changes in disease, predominantly because of increasing non-myocytes numbers and diversity. The increasing abundance of a cell type may be misinterpreted as upregulation of a gene in cardiac tissue, even if said gene is stably expressed (or, conceivably, even reduced) in a given cell population [106]. All this shows that gene expression analysis from tissue may not only be less sensitive, but even lead to false conclusions [106].

The most suitable experimental approach to study cell type-specific gene expression should be chosen in accordance with the scientific question at hand. scRNA-seq is now widely accessible and frequently used in cardiovascular science [60, 118]. One key strength of single-cell technologies is their suitability for assessing cell type diversity and cell-to-cell gene expression variability [29]. In addition, the power of the analysis can be increased by including spatial information, and/or multiple time points during development, disease progression, or therapy [10, 95, 100, 111]. Single-cell techniques enable researchers to determine gene expression in all captured cells, thereby reducing selection bias. However, some experimental factors that influence results need to be considered. Before sequencing, heart tissue is dissociated into single cells. This preparation of single-cell suspensions, usually achieved by a combination of enzymatic and mechanical disruption [185], is a critical step because it has a strong impact on obtaining a reproducible yield of viable cells that reflects the composition of cell populations in the heart, especially in diseased tissue with excessive presence of ECM. In addition, some of the available microfluidic or droplet-based scRNA-seq systems are not suitable for large cells such as cardiomyocytes [60]. In contrast to intact cells, nuclei are of small size and may be isolated for transcriptomic and epigenetic studies from fresh or frozen heart tissue without prior enzymatic digestion [8, 157]. Thus, researchers in the cardiovascular field are increasingly relying on snRNA-seq [60, 102, 188]. Despite improved low RNA input protocols the number of transcripts per cell, and the even lower number per nucleus, limits the number of genes that can be detected, cutting off low abundant transcripts and potentially making snRNA-seq more prone to noise [29]. In addition, rare cell types may only be represented by a few cells. Here, bulk RNA-seq may have advantages for determining differential gene expression in a defined cell population, as long as that population is well represented and identifiable in post-isolation cell suspensions. Cells or nuclei can be isolated by flow cytometry or magnetic bead-assisted sorting, based on their size, surface marker expression, or genetic labelling [58, 96, 105, 106, 158].

Cell culture has been used for decades to study cellular functions in a defined and reproducible setting. More recently, more complex approaches for assessing heterocellular interactions ex vivo have been developed, ranging from multi-cell type and spatially structured co-cultures and 3D organoids to printed cell and ECM scaffolds or ‘heart-on-a-chip’ systems [31, 87, 119, 124]. While 3D organoids largely rely on the self-assembly of stem cells into spheroids, 3D printed cell assemblies are typically built from defined mixtures of cardiac cell types and exogenous extracellular matrix [124]. These systems may be adjusted in multiple ways to meet different experimental needs. Generally, one can say that systems that model more mature tissue are more difficult to scale for high-throughput applications, which requires careful balancing of ease of utilization with the level of insight gained [31]. An alternative to engineered tissue models are living myocardial tissue slices [23, 175, 206, 207]. These slices offer an interesting addition to the tool-kit of heterocellular interaction research [151, 175], as live tissue can be kept for days, even weeks [175], and it is possible to isolate well-preserved cells from the same tissue donor on multiple days [211]. This opens the door to the exploration of intervention effects, such as exposure to modulators of biochemical of biophysical signalling [150], in as far as they can be achieved outside the body (i.e. in the absence of circulating cells of extra-cardiac origin) [63].

The perhaps most important breakthrough in studying heterocellular interactions has come from the availability of optogenetic tools to target specific cell populations, whether with effectors that allow one to modulate function of target cells by exposure to light [76], or with reporters of cell activity that allow one to explore minority cell contributions to ensemble activity that may be dominated by another cell type (e.g. fibroblast electrophysiology signals, which would otherwise be drowned-out by the dominating electrophysiological activity of cardiomyocytes [160]. The availability of effectors and reporters is continuously growing [16] including light-activated enzymes (such as Cre) that allow one to steer gene expression not only in a cell type-specific setting, but also in a spatio-temporally controlled manner [213].

### …for therapeutic interventions

**Fig. 4:**
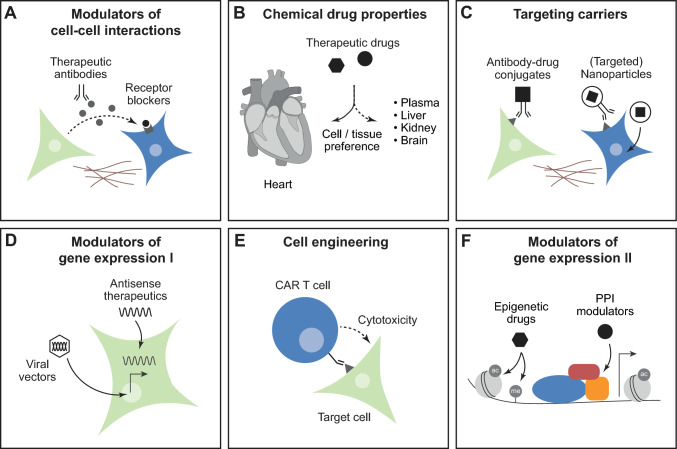
Implications for therapeutic interventions. **A** Biochemical cell–cell interactions via secreted molecules can be inhibited by therapeutic antibodies or receptor blockers. **B** Chemical properties influence tissue and cell distribution of therapeutic drugs. **C** Targeting carriers such as antibody–drug conjugates or nanoparticles with or without modified surface antigens can steer drug delivery towards certain cell types. **D** Antisense therapeutics and viral vectors enable gene silencing, overexpression, and DNA editing in cardiac cells. **E** Chimeric antigen receptor (CAR) T cells directed against a specific target cell type can be obtained by genetic engineering. **F** Pathological gene expression can be inhibited by epigenetic drugs or modulators of protein–protein interaction (PPI)

A more detailed understanding of cell type-specific molecular functions and cellular cross-talk from basic research may enable new therapeutic approaches in cardiovascular disease (Fig. 4). Many drugs that are currently used in cardiovascular medicine, such as betablockers, angiotensin receptor blockers, or endothelin receptor antagonists, are used to modulate biochemical cell–cell interactions that are mediated via secreted factors, such as hormones, cytokines, or growth factors (Fig. 4A). This includes long-range interactions via circulating factors, as well as local paracrine actions. Cell–cell interactions can be modulated in multiple ways, e.g. by receptor block or by preventing enzymatic activity. For example, the combined angiotensin receptor and neprilysin inhibitor sacubitril/valsartan prevents the degradation of natriuretic peptides that are secreted by cardiomyocytes, thereby increasing their local and systemic levels. Natriuretic peptides act on multiple organ systems including the kidney or the vasculature, but they also have direct anti-hypertrophic and anti-fibrotic effects on the heart [108]. More recently, neutralizing antibodies have been developed to eliminate secreted factors and prevent their effects on cardiac cells. This principle has been applied therapeutically, e.g. for the interleukin-1 targeting antibody canakinumab [223], but needs to be considered with care for other indications due to potential risks of adverse effects on the cardiovascular system, e.g. for VEGF targeting antibodies [194]. The difficulty of targeting factors to a specific organ or cell type, combined with the lack of specificity for the often pleiotropic actions of secreted factors, is a common issue with therapeutics targeting cell–cell interactions.

Cell type-selective pharmacology may be one way to enhance efficacy of treatments, while reducing undesired side-effects [33]. Effective concentrations of therapeutic drugs depend on their resorption, tissue penetration, metabolism, and elimination. Circulating drugs have to pass multiple barriers before they reach their targets, including vessel walls, ECM, and plasma membranes of target cells [222]. Several therapeutics, including antiarrhythmic drugs, show higher concentrations in heart tissue than in plasma [198], however, this may also be true for other organs and does not necessarily indicate organ-specificity (Fig. 4B).

A number of strategies for drug targeting share the idea of linking a therapeutic ‘cargo’ to a cell-selective ‘carrier’ [222] (Fig. 4C). Possible cargos include not only small molecules or peptide drugs, but also nucleic acid therapeutics or antibodies. Potential carriers are antibodies against a specific epitope, peptides or small molecules binding to surface receptors or extracellular matrix components, as well as liposomes [22, 178, 222]. Development and clinical application of targeted drug delivery systems is most advanced in oncology [22, 222], while suitable target structures for cardiac cells remain to be exploited. Based on the findings described above, CD36 appears to be a promising candidate, however, this has not been experimentally tested yet. In addition, certain differentially expressed surface markers may lead to preferential binding of substances, such as lectins. Lectin A (a virulence factor that promotes endocytosis of bacteria into host cells), for example, binds to cardiac non-myocytes but not to muscle cells, sensitizing the former to mechanical activation [37]. Similarly, regional cell specificity may arise from targeting differentially expressed proteins, such as ion channels. Attempts to develop atrial cardiomyocyte-selective drugs for atrial fibrillation have not, however, been very successful—in part because atrial preferentiality, let alone ‘selectivity’, established in healthy tissue, may not be sustained after disease-induced electrophysiological remodelling [173].

RNA-based therapy represents another innovative approach that is currently moving forward (Fig. 4D) [169]. Most advanced is the development of antisense oligonucleotides and small interfering ‘siRNA’ molecules, with first therapeutics approved for clinical use—most of them for treatment of hereditary diseases [212]. In the cardiovascular field, the siRNA formulations patisiran [2] and inclisiran [164] are used for treatment of transthyretin amyloidosis or uncontrolled hypercholesterolaemia in atherosclerosis, respectively. However, these siRNA primarily target the liver, not cardiac cells [212]. Recently, a phase 1 clinical trial in patients with chronic heart failure demonstrated beneficial effects of an antisense oligonucleotide against miR-132 [193]. Mechanistic studies had shown before that miR-132 is a driver of pathological hypertrophy, expressed in cardiomyocytes but not in fibroblasts [200].

Viral vectors, most commonly adeno-associated viral (AVV) vectors, are used for gene transfer in basic cardiovascular research, and they have been tested for gene therapy in clinical trials [168] (Fig. 4D). Viral vectors bind to surface receptors of their target cells, undergo endocytosis and intracellular processing, and release their genetic information into the nucleus [219]. Depending on their capsid structure, different viral vectors or serotypes may preferentially target different tissues and cell types, with AAV1, AAV6, AAV8, and AAV9 showing high efficacy in transducing cardiomyocytes [168]. To improve tropism towards certain cell types, capsid modification can be a productive [170]. Cell type-specific promoters can be used to limit gene expression to the cell type of interest, e.g. TCF21 or periostin to target (myo-)fibroblasts [54, 149], potentially even in a heart chamber specific manner [18]. Viral vectors are mostly used to directly increase or restore the expression of proteins that are involved in cardiac function [168]. More recently, genome editing tools such as CRISPR–Cas9 [103] have been applied successfully in preclinical models [122, 138] with the long-term vision of treating hereditary cardiac disease that are caused by single gene mutations. Furthermore, a catalytically inactive Cas9 (dCas9) can be fused to histone acetyltransferases, histone deacetylases, or DNA methyltransferases and transferred by viral vectors, which could enable targeted epigenetic modulation of gene expression [103].

Chimeric antigen receptor (CAR)-T cells are genetically engineered T cells, directed against a specific antigen on the surface of their target cell [97] (Fig. 4E). CAR-T cells have been successfully used to tackle cardiac fibrosis in mice [4, 173]. Cardiac fibroblasts were selectively destroyed by CAR-T cells aiming at fibroblast activation protein (FAP) [4, 173]. In most studies, T cells were genetically modified by adenoviral transduction ex vivo, requiring extensive lab work and impeding clinical use. Very recently, a protocol has been reported to generate FAP-CAR-T cells in vivo*.* The authors used lipid nanoparticles that were decorated with anti-CD5 antibodies, targeting T cells and carrying mRNA cargo, directing the T cell against FAP [173]. This example illustrates how knowledge of cell type-specific features can facilitate targeted therapies. However, despite these promising results, substantial barriers remain, related to immunogenicity, delivery, and specificity of RNA-based or viral therapies, that need to be addressed before routine use in humans [168, 212].

Epigenetic drugs such as histone deacetylase (HDAC) or bromodomain and extra-terminal motif inhibitors show therapeutic potential in heart disease [45, 129, 197, 208] (Fig. 4F). Cell type-specific gene expression is determined by the activity of regulatory elements (‘enhancers’ or ‘suppressors’) that are characterized by posttranslational modification of neighbouring histones and low methylation of the DNA. Enhancers are primed by pioneering transcription factors in a cell type-specific manner, making them accessible to signal-dependent transcription factors [68]. DNA methylation-guided analysis of cardiomyocytes, fibroblasts, endothelial cells, and macrophages revealed that approximately 40% of the regulatory elements are specific for one of those cell types [106]. An extended analysis of accessible chromatin by snRNA-seq of nine different cardiac cell types identified ~ 6% specific regulatory elements, which were associated with typical cell functions [73]. The cell-specificity of enhancers makes them interesting potential drug targets. Therapeutic drugs such as HDAC inhibitors will potently be able to inhibit gene expression [129]. However, strategies need to be developed to steer epigenetic inhibitors towards specific genomic regions or cell types. Another approach to steer gene expression is to modulate protein–protein interactions within nuclear transcription factor complexes [33]. For instance, the novel non-steroidal mineralocorticoid receptor antagonist finerenone acts as a ‘bulky’ antagonist, mediating a conformational change of the receptor that impedes interaction with certain cofactors [88]. It has been suggested that the resulting distinct cofactor recruitment and ligand-specific gene program may contribute to an enhanced anti-fibrotic activity of finerenone compared, to other mineralocorticoid receptor antagonists [64] (Fig. 4F).

## Conclusions

The heterocellular nature of the heart has important implications for basic and translational research in cardiology. In addition to cardiomyocytes, the heart consists of endothelial cells, smooth muscle cells, fibroblasts, various immune cells, and other cell types including pericytes, adipocytes, neurons, and Schwann cells. The advent of scRNA-seq technologies, which enable the detection of subtle distinctions in cellular identities, has brought some of the not so usual suspects more into focus. However, it has also challenged the traditional view of what defines a cell type. Researchers need to consider the distinct molecular, biochemical, and biophysical properties of different cell types in the heart that require specific experimental approaches. A better understanding of cell type-specific molecular functions and cellular interactions, arising from basic research, may lead to new therapeutic approaches for cardiovascular medicine. Currently, many drugs used in cardiovascular medicine aim to modulate biochemical cell–cell interactions that are mediated via secreted factors. However, the lack of specificity for the often pleiotropic actions of these secreted factors is a common challenge. Several strategies for therapeutic targeting have been developed, including the linkage of a drug to a cell-selective ‘carrier’, RNA-based therapeutics, epigenetic drugs, or CAR-T cells. Novel approaches such as optogenetics, currently in experimental use for cell type-specific steering of cardiac function by exposure to light, may progress to clinical development in the future—in particular if ‘single-exposure’ to light (via catheter) can give rise to sustained effects on the heart (e.g. triggering local and cell type-specific expression of certain traits). Overall, it becomes increasingly clear that the cardiomyocyte-centric view of the heart is obsolete, while the identification of heterocellular identities and interactions promises to have substantial implications for the future of cardiology.

## Abbreviations

αSMA: α Smooth muscle actin
CCR2: C–C chemokine receptor 2
DDR2: Discoidin-domain receptor 2
ECM: Extracellular matrix
ET: Endothelin
FAP: Fibroblast activation protein
HDAC: Histone deacetylase
NG2: Neuron–glial antigen 2
RNA: Ribonucleic acid
RNA-seq: RNA sequencing
scRNA-seq: Single cell RNA-seq
snRNA-seq: Single nucleus RNA-seq
siRNA: Small interfering RNA
T-tubulues: Transverse tubules
VEGF/VEGFB: Vascular endothelial growth factor/vascular endothelial growth factor B
